# Patient perspectives on practice guidance in nursing education: a scoping review

**DOI:** 10.1186/s12909-026-09019-8

**Published:** 2026-03-19

**Authors:** Andrea Glodek, Bettina Dauer, Bernd Reuschenbach

**Affiliations:** 1 Dept. of Health and Nursing, Catholic University of Applied Science, Preysingstr. 95, Munich, D-81667 Germany; 2 Federal Institute for Vocational Education and Training (BIBB), Friedrich-Ebert-Allee 114-116, Bonn, D-53113 Germany

**Keywords:** Teaching rounds, Patient participation, Patient rights, Patient satisfaction, Education, Nursing, Patient-centered care, Nursing education, Nursing practice, Clinical teaching, Patient involvement

## Abstract

**Background:**

Practical training in health care involves caring for patients in hospitals, long-term care facilities, and outpatient settings. This scoping review examines patients’ perspectives on their involvement in clinical teaching and explores how evidence from medical education can guide nursing education when research is lacking.

**Methods:**

A systematic search of PubMed, CINAHL, and LIVIVO was conducted. Studies published in German and English that addressed clinical practice education in nursing and medicine were eligible.A total of 1,984 articles were screened based on the inclusion and exclusion criteria, of which 45 met the established criteria. Because no nursing-specific studies were identified during the initial search, the review was expanded to include medical education literature. This decision was based on the parallels between nursing and medical training, particularly regarding clinical competencies and patient-centred care.

**Results:**

The 45 extracted records provided findings on five topics: (1) Motivations for participation: Patients reported both altruistic motives (contributing to education) and personal benefits (increased attention and educational value). Acceptance rates ranged from 78–100% in most studies. (2) Barriers to participation: Privacy concerns dominated, particularly for intimate examinations, with strong gender-concordance preferences. Concerns about competence and cultural/religious factors also influenced willingness. (3) Patient experiences: Experiences were generally positive; however, physical discomfort, inadequate supervision, and boredom were reported. (4) Information and consent: Many patients lacked awareness of teaching contexts, role hierarchies, and their right to refuse participation, with some reporting feelings of coercion. (5) Organizational factors: Small group sizes, appropriate timing, and clear role communication enhanced patient experiences.

**Conclusions:**

No nursing-specific evidence is available on patient perspectives on clinical teaching. While medical education findings suggest that patients generally accept student involvement when privacy, communication, and consent are respected, the direct applicability to nursing contexts—particularly in outpatient, long-term care, and home settings—remains unvalidated. Nursing-specific research examining patient perspectives across diverse care settings is urgently needed to develop ethical, evidence-based guidelines for clinical education.

**Supplementary Information:**

The online version contains supplementary material available at 10.1186/s12909-026-09019-8.

## Background

Clinical training in health professions requires patients’ informed consent and the acceptance of learner involvement in their care. The incorporation of patients’ perspectives into clinical education is essential for upholding ethical principles—particularly autonomy and informed consent—and for ensuring patient-centred, high-quality care [1–3].

Fundamental clinical competencies can be acquired only through direct patient engagement, making patient participation an indispensable component of professional education. Bedside teaching, defined as “clinical teaching in the presence of the patient, usually with a three-way relationship between clinical teacher, student and the patient” [4], represents a cornerstone of education in both nursing and medicine.

Understanding the factors that influence patients’ willingness to accept or refuse student involvement is essential for ethical practices and positive educational experiences [5]. Conceptualizing patients as active partners rather than as passive recipients—through informed consent, transparent communication, and respect for preferences—is fundamental to patient-centred education. Patient-centred care constitutes a core ethical principle of nursing, as reflected in the ICN Code of Ethics for Nurses (2021), which explicitly extends its application to nursing education and supervision contexts [6]. However, systematic attention to patients’ experiences during clinical teaching remains limited. While extensive literature addresses learner assessment by educators [7, 8], the actual patient perspective during this learning process has received insufficient attention. This evidence gap is particularly urgent: nursing education lacks discipline-specific guidelines grounded in patient experience, and ethical clinical practice requires that patient voices are systematically heard and integrated across all care settings. Without dedicated nursing-specific research, clinical educators risk applying medical education evidence uncritically to fundamentally different care contexts.

This scoping review examines patients’ perspectives on and experiences with student participation in clinical teaching. A preliminary literature search revealed no nursing-specific studies addressing this topic, necessitating the inclusion of relevant studies from medical education. This approach is justified by the substantial parallels between nursing and medical education: both involve supervised patient care in clinical settings, employ similar workplace-based pedagogical approaches, require comparable interpersonal and communication competencies, and emphasize patient-centred practice principles. Nursing encompasses broader psychosocial and daily living support, whereas medicine focuses primarily on diagnosis and treatment. Nevertheless, differences in professional roles, the scope of practice, and educational structures require careful interpretation when evidence is transferred across contexts.

This review is aimed at (1) identifying key themes in patient perspectives on student involvement in clinical teaching and assessment activities, (2) synthesizing existing evidence from medical education, (3) assessing the potential applicability and transferability of this evidence to nursing education contexts, and (4) proposing directions for nursing-specific research on patient perspectives in clinical education.

## Methods

### Search strategy

A systematic search strategy using predefined search terms and keywords was developed [9, 10]. The protocol is available from the authors upon request. The databases PubMed, Cumulative Index to Nursing and Allied Health (CINAHL), and LIVIVO were selected to cover international and Anglo-American medical and nursing literature, international nursing literature, and German-speaking and European sources, respectively. The literature search was conducted in August 2022. Given the exploratory nature of the topic and the limited existing research, no date restrictions were applied. The searches were limited to English and German language publications.

The search terms were constructed using the Population-Concept-Context (PCC) framework [11]: that is, the population (patients and care recipients), concept (attitudes, preferences, satisfaction, perspectives, and experiences), and context (bedside teaching, clinical teaching, and practice education) framework. Boolean operators and database-specific subject headings (MeSH terms for PubMed; CINAHL headings) were used. The complete search strategy is detailed in Additional file 1 (see Additional file 1). The initial search yielded 1,984 records. After the removal of the 297 duplicates, 1,687 records were reviewed for title and abstract screening. Among these, 76 articles were identified using a bibliography.

### Study selection

Following the PRISMA-ScR guidelines [9], two reviewers (AG, BD) independently screened titles and abstracts, followed by full-text screening of potentially eligible articles using Covidence software (Veritas Health Innovation, Melbourne, Australia). Disagreements were resolved through consensus discussion. A third reviewer was available for adjudication but was not needed. Articles in which patients were asked for their views on practical instruction or bedside teaching or that systematically compiled these views were included.

The inclusion criteria were as follows: studies published in German or English; empirical studies or systematic reviews in which patients or care recipients were directly asked about their perspectives on, attitudes towards, preferences regarding, or experiences with student involvement in clinical teaching or bedside teaching; studies set in nursing or medical education contexts (inpatient, outpatient, long-term care, or community settings); and studies that systematically reported or compiled patient views.

The exclusion criteria were as follows: patient interaction outside formal education situations without a teacher, rounds without explicit teaching, patient participation in continuing education and training, studies involving training patients in simulated teaching units, studies examining patient handoffs, studies without educational focus and studies from disciplines other than nursing or medicine or involving nonhuman subjects.

**Fig. 1:**
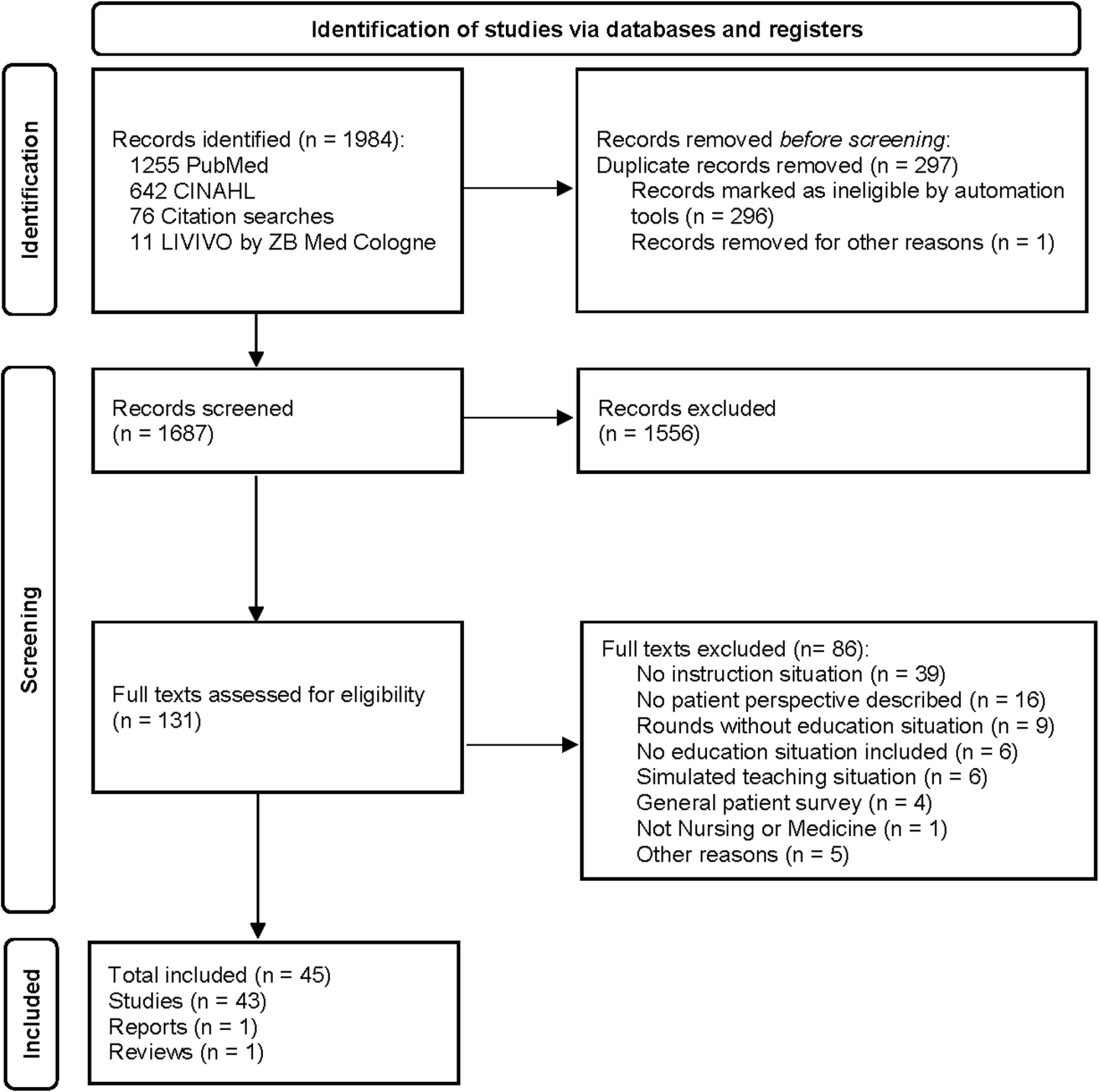
Flow diagram of study selection process, PRISMA 2020

For full-text screening, 131 articles were included. Of these 86 articles were excluded for the following reasons: no educational context (*n* = 39), no patient perspective described (*n* = 16), rounds without educational situation (*n* = 9), no education situation included (*n* = 6), simulated teaching situations (*n* = 6), a general patient survey without education focus (*n* = 4), a lack of nursing or medicine (*n* = 1), and other reasons (*n* = 5) (see Fig. 1). A total of 45 articles, including 37 quantitative surveys, six qualitative interviews, one systematic review, and one expert commentary, met the established criteria for this review.

### Data extraction

Two researchers (AG and BD) collaboratively developed and pilot-tested a standardized data extraction form. Two reviewers then independently extracted data from all the included studies. The extracted data included the study characteristics (author, year, and country), methodological details (design and research objectives), participant information (sample size and demographics), and key findings related to patient perspectives, attitudes and experiences related to student involvement in clinical care. Discrepancies were identified and resolved through discussion until a consensus was reached. The data extraction form is provided in Table 1 (see Table 1).

**Table 1:** Article with characteristics

Author	Title	Authors	Country	Professions	Objectives	Method
Abdulghani et al. [12]	Patient attitudes toward medical students in Riyadh, Saudi Arabia.	Abdulghani, HM; Al-Rukban, MO; Ahmad, SS.	Other: Saudi Arabia	Medicine	(1) To assess patients’ attitudes toward involving medical students in their care in a teaching and a public general hospital; (2) To assess factors that may affect patients’ opinions toward their right to refuse medical students and their preferences for medical student involvement and the role of medical students.	Questionnaire
Alawad and Younis [13]	Patients’ attitude towards undergraduate medical students at university charity teaching hospital in Sudan.	Alawad, AAM; Younis, FH.	Other: Sudan	Medicine	To explore the attitudes of patients towards medical students at a University charity teaching hospital, and to explore the determinants of those attitudes.	Questionnaire
Aljoudi et al. [14]	Patients’ attitudes towards the participation of medical students in clinical examination and care in Western Saudi Arabia.	Aljoudi, SB; Alsolami, SS; Farahat, FM; Alsaywid, B; Abuznadah, W.	Other: Saudi Arabia	Medicine	To assess patient attitudes and associated factors related to medical student involvement in their care.	Questionnaire
Anfinan et al. [15]	Obstetric and gynecologic patients’ attitudes and perceptions toward medical students in Saudi Arabia.	Anfinan, N; Alghunaim, N; Boker, A; Hussain, A; Almarstani, A; Basalamah, H; Sait, H; Arif, R; Sait, K	Other: Saudi Arabia	Medicine	To identify patients’ attitudes, preferences, and comfort levels regarding the presence and involvement of medical students during consultations and examinations.	Questionnaire
Arolker et al. [16]	‘They’ve got to learn’--a qualitative study exploring the views of patients and staff regarding medical student teaching in a hospice.	Arolker, M; Barnes, J; Gadoud, A; Jones, L; Barnes, L; Johnson, MJ.	The UK	Medicine	To explore and compare the views of hospice patients and healthcare staff (doctors and nurses) about patient involvement in medical student teaching.	Interviews
Ben Salah et al. [17]	Patients’ attitude toward bedside teaching in Tunisia.	Ben Salah, A; El Mhamdi, S; Bouanene, I; Sriha, A; Soltani, M	Other: Tunisia	Medicine	(1) To assess patients’ attitudes toward the presence of medical students in a teaching hospital; (2) To identify factors that may influence these attitudes in order to address them with a view to improving bedside teaching.	Questionnaire
Bukhari et al. [18]	Patients’ attitude toward medical students rotating in the dermatology clinic.	Bukhari, I; AlAkloby, Omar; Al Saeed, W	Other: Saudi Arabia	Medicine	To examine patients’ attitude towards medical students at the dermatology clinics in Saudia Arabia.	Questionnaire
Callaly et al. [19]	Is the Irish bedside best?	Callaly, EL; Yusra, M; Sreenan, S; McCormack, P	Other: Ireland	Medicine	Survey of inpatient attitudes toward bedside teaching for undergraduate medical students.	Questionnaire
Carey et al. [4]	Validation of a questionnaire exploring patient attitudes toward bedside teaching.	Carey, MO; O’Riordan, N; Carty, M; Ivers, M; Taylor, LK; Higgins, MF.	Other: Ireland	Medicine	Validation of a questionnaire to investigate patients’ attitudes toward bedside teaching.	Questionnaire
Carty et al. [20]	Patient perspectives of bedside teaching in an obstetrics, gynaecology and neonatology hospital.	Carty, Michelle; O’Riordan, Nicola; Ivers, Mary; Higgins, Mary F.	Other: Ireland	Medicine	Qualitative study exploring patients’ experiences and opinions on bedside teaching.	Interviews
Ching et al. [21]	Factors influencing obstetric and gynecologic patients’ decisions toward medical student involvement in the outpatient setting.	Ching, SuL.; Gates, EA; Robertson PA	United States	Medicine	To identify factors involved in patient acceptance or refusal of medical student participation in their outpatient obstetric-gynecologic visit.	Questionnaire
Choudhury et al. [22]	Patients’ attitudes toward the presence of medical students during consultations.	Choudhury, TR; Moosa, AA; Cushing, A; Bestwick, J	The UK	Medicine	To examine the factors, particularly ethnicity, that influence patients’ attitudes toward medical students.	Questionnaire
Chretien et al. [23]	A qualitative study of the meaning of physical examination teaching for patients.	Chretien, KC; Goldman, EF; Craven, KE; Faselis, CJ	The United States	Medicine	Survey on the importance of teaching about the physical examination from the perspective of patients.	Interviews
Figueiró-Filho et al. [24]	Minimal supervision out-patient clinical teaching.	Figueiró-Filho, EAn; Amaral, E; McKinley, D; Bezuidenhout, J; Tekian A	Other: Brazil, The United States, South Africa	Medicine	Patients’ and medical students’ views of clinical teaching with minimal supervision in an ambulatory care setting. Evaluation of student performance from the patient’s perspective.	Questionnaire
Franks and Rudd [25]	Medical student teaching in a hospice - What do the patients think about it?	Franks, A; Rudd, N	The UK	Medicine	Hospice patients’ feelings about taking part in undergraduate teaching activity.	Questionnaire
Gebrekirkos and Van Wynk [26]	Effect of bedside teaching activities on patients’ experiences at an Ethiopian hospital.	Gebrekirkos, FA; van Wyk, JM	Other: Ethiopia, South Africa	Medicine	To explore the perceptions and attitudes of patients towards the clinical teaching process.	Questionnaire
Al Ghobain et al. [27]	Patients’ perceptions towards the participation of medical students in their care.	Al Ghobain, MA; Alghamdi, A; Arab, A; Alaem, N; Aldress, T; Ruhyiem, M	Other: Saudi Arabia	Medicine	To investigate patients’ perceptions of the participation of medical students in their care.	Questionnaire
Gillon et al. [28]	Is the traditional ‘teaching ward round’ appropriate in a palliative care unit?	Gillon, S; Ward, J; Hustwit, J; Taylor, S	The UK	Medicine	To examine the number of staff and students present in consultant-led hospice ward rounds.	Questionnaire
Harris et al. [29]	Should we involve terminally ill patients in teaching medical students? A systematic review of patient’s views.	Harris, DG; Coles, B; Willoughby, HM.	The UK	Medicine	Systematic review of available published research that has examined how terminally ill patients perceive participation in undergraduate medical education.	Review
Hartz and Beal [30]	Patients’ attitudes and comfort levels regarding medical students’ involvement in obstetrics-gynecology outpatient clinics.	Hartz, BM; Beal, JR	The United States	Medicine	To identify the views of obstetrics-gynecology patients on the roles of medical students.	Questionnaire
Iqbal et al. [31]	Participation of medical students in patient care: How do patients perceive it?	Iqbal, MZ; Bukhamsin, EY; Alghareeb, FY; Almarri, NM; Aldajani, LM; Busaleh, HA	Other: Saudi Arabia	Medicine	Assessment of patients’ perceptions and views regarding acceptance of medical students in a Saudi provincial hospital.	Questionnaire
Khoo et al. [32]	Parents’ perceptions of bedside teaching.	Khoo, EJ; Parameshwara, N; Kutzsche, S	Other: Malaysia	Medicine	To examine the perceptions of parents who participated with their children in bedside teaching of medical students.	Interviews
Kianmehr et al. [33]	Medical student and patient perspectives on bedside teaching.	Kianmehr, N; Mofidi, M; Yazdanpanah, R; Ahmadi, MA	Other: Iran	Medicine	To examining student and patient perspectives on bedside teaching.	Questionnaire
Klaber and Pollock [34]	Clinical teaching in paediatrics: understanding perceptions, motives and concerns.	Klaber, RE; Pollock, I	The UK	Medicine	Research on the views of children and young people, their parents, and trainees and tutors on why children and their families participate in courses as subjects of instruction.	Questionnaire
Koehler and McMenamin [35]	Would you consent to being examined by a medical student? Western Australian general public survey.	Koehler, N; McMenamin, C	Australia	Medicine	To determine the public’s perception of various examination by medical students for teaching purposes only.	Questionnaire
Kriesen et al. [36]	Perception of bedside teaching within the palliative care setting-views from patients, students and staff members.	Kriesen, U; Altiner, A; Müller-Hilke, B	Other: Germany	Medicine	(1) To analyze perceptions of bedside teaching in a palliative care unit from the perspectives of students, staff, and patients; (2) To define prerequisites for effective and patient-centered bedside teaching in palliative care units.	Interviews
Kuan and O’Donnell [37]	Medical students in the emergency department: how do patients view participation in clinical teaching?	Kuan, S; O’Donnell, JJ.	Other: Ireland	Medicine	Patients’ acceptance of medical students and their attitudes toward participation in clinical teaching.	Questionnaire
Khan et al. [38]	Patients’ receptiveness for medical students during consultation in out patient department of a teaching hospital in Karachi Pakistan.	Laiq-uz-Zaman Khan, M; Jawaid, M; Hafeez, K	Other: Pakistan	Medicine	Patient satisfaction with treatment and care, when medical students were present compared with when no medical students were present.	Questionnaire
Law et al. [39]	The effect of clinical teaching on patient satisfaction in rural and community settings.	Law, M; Hamilton, M; Bridge, E; Brown, A; Greenway, M; Stobbe, K	Canada	Medicine	To measure patient satisfaction and factors influencing patients’ decisions to involve medical students in their care.	Questionnaire
Lucas and Pearson [40]	Patient perceptions of their role in undergraduate medical education within a primary care teaching practice.	Lucas, B; Pearson, D	The UK	Medicine	To explore patients’ views of their role in undergraduate medical education in an outpatient educational practice setting.	Interviews
Lucas et al. [41]	Patient motivation to participate in medical education.	Lucas, B; Wiegand, S; Jahn, O; Greiner, F; Walcher, F; Piatek, S	Other: Germany	Medicine	To determine patient willingness to participate in medical teaching and consent to produce multimedia educational content.	Questionnaire
Marwan et al. [42]	Are medical students accepted by patients in teaching hospitals?	Marwan, Y; Al-Saddique, M; Hassan, A; Karim, J; Al-Saleh, M	Other: Kuwait	Medicine	To examine patient acceptance of medical students in teaching hospitals as a function of sociodemographic characteristics.	Questionnaire
McKimm [43]	Involving patients in clinical education.	McKimm, J	Other: New Zealand	Nursing; medicine	A review article on the scope, principles, and practice of patient involvement in clinical education and on the application of best practices in patient involvement.	Text
Monnickendam et al. [44]	Patients’ attitudes toward the presence of medical students in family practice consultations.	Monnickendam, SM; Vinker, S; Zalewski, S; Cohen, O; Kitai, E	Other: Israel	Medicine	To investigate patients’ attitudes and expectations towards participation in teaching general practice, and the influence of a student’s presence on the consultation.	Questionnaire
Mora-Pinzon et al. [45]	What do patients think of medical students during their hospitalization? One institution’s experience.	Mora-Pinzon, M; Lal, A; Edquist, S; Fracescatti, A; Hughes, T; Hayden, D; Brand, M; Saclarides, T	The United States	Medicine	To explore patient attitudes toward medical students in the inpatient setting and the factors that may influence them.	Questionnaire
Mosalanejad et al. [2]	A holistic approach to bedside teaching from the views of main users.	Mosalanejad, L; Hojjat, M; Gholami, M	Other: Iran	Nursing; medicine	To examine the quality and effectiveness of bedside teaching from the perspectives of students, faculty, and patients.	Questionnaire
Nair et al. [46]	Student and patient perspectives on bedside teaching.	Nair, BR; Coughlan, JL; Hensley, MJ.	Australia	Medicine	To explore the opinions of patients, students, interns, and residents about bedside teaching.	Questionnaire
O’Flynn et al. [47]	Consent and confidentiality in teaching in general practice: survey of patients’ views on presence of students.	O’Flynn, N; Spencer, J; Jones, R	The UK	Medicine	Survey of patients’ views on consent and confidentiality.	Questionnaire
Onotai et al. [48]	Patients’ perception and attitude towards medical students’ involvement in patients care at a Nigerian University Teaching Hospital.	Onotai, LO; Asuquo, EO; Amadi, E; Amadi-Oparelli, A; Ali, DU	Other: Nigeria	Medicine	To determine the overall attitude and perception of patients toward medical students’ involvement in their hospital care.	Questionnaire
Petersen et al. [49]	A randomized controlled study comparing educational outcomes of examination room versus conference room staffing.	Petersen K; Rosenbaum ME; Kreiter CD; Thomas A; Vogelgesang SA; Lawry GV.	The United States	Medicine	To examine patient, learner, and faculty perceptions in an outpatient teaching setting.	Questionnaire
Rizk et al. [50]	Women’s perceptions of and experiences with medical student involvement in outpatient obstetric and gynecologic care in the United Arab Emirates.	Rizk, DE; Al-Shebah, A; El-Zubeir, MA; Thomas, LB; Hassan, MY; Ezimokhai, M	Other: United Arab Emirates	Medicine	To determine women’s perceptions, experiences, satisfaction and comfort level of medical students participating in their health care.	Questionnaire
Rockey et al. [51]	Patient participation in medical student teaching: a survey of hospital patients.	Rockey, NG; Ramos, GP; Romanski, S; Bierle, D; Bartlett, M; Halland, M	The United States	Medicine	This study investigated inpatients’ willingness, motivations and experience with participation in medical student bedside teaching.	Questionnaire
Rubliauskas et al. [52]	Patient feedback on medical students in tertiary health care: are medical students accepted in clinical practice?	Rubliauskas, K; Šalkauskaitė, A; Macas, A	Other: Lithuania	Medicine	To determine patient attitudes toward the presence of medical students during surgery and the administration of anesthesia.	Questionnaire
Sayed-Hassan et al. [53]	Patient attitudes towards medical students at Damascus University teaching hospitals.	Sayed-Hassan, RM; Bashour, HN; Koudsi, AY.	Other: Syria	Medicine	To determine Syrian patients’ attitudes toward the presence of medical students, their comfort level during the examination, and their preferences regarding medical student participation.	Questionnaire
Shetty et al. [54]	Patient outlook on bedside teaching in a medical school.	Shetty, PA; Magazine, R; Chogtu, B	Other: India	Medicine	To evaluate the views of diverse groups of patients on bedside teaching and the degree of involvement of medical students in their clinical decision-making processes.	Questionnaire

### Data synthesis

The extracted findings were synthesized using inductive thematic analysis. Two reviewers independently coded the patient-reported findings and collaboratively developed preliminary thematic categories through iterative comparison and discussion. Categories were refined through multiple team meetings until consensus was achieved. To enhance trustworthiness, an independent expert in clinical education research (affiliation provided in the acknowledgements) reviewed the thematic framework and confirmed its relevance and coherence.

## Results

The 45 included studies [2, 4, 12–54] collectively enrolled 9,538 patients. All the studies examined medical education contexts; no nursing-specific studies were identified. The study designs included quantitative surveys (*n* = 37), qualitative interviews (*n* = 6), one systematic review, and one expert commentary. The participant populations varied considerably. Several studies included paediatric populations and their parents: 26 children and adolescents (10–17 years) and 54 parents [34], 56 children under two years old with parents and 53 children over two years old with parents [42], 30 children under six years old and their parents [17], and 54 parents [32]. Researchers on one study [2] adopted a multi-perspective approach, surveying both patients and medical/nursing students (*n* = 70). Given that patient perspectives on medical education were surveyed almost exclusively, the findings are presented without distinguishing between nursing and medical education contexts, although the implications for nursing education are discussed later.

Five overarching themes emerged: (1) patient motivations for participation, (2) barriers to and reasons for refusal, (3) patient evaluations of clinical education experiences, (4) information provision and consent processes, and (5) educational and organizational context factors (see Table 2).

**Table 2:** Articles with results of data extraction

Study ID	Participants	Sample size	patient motivations for participation	barriers to and reasons for refusal	patient evaluations of clinical education experiences	information provision and consent processes	educational and organizational context factors
Abdulghani et al. [12]	inpatients and outpatients in various teaching hospitals in Saudi Arabia	492 patients		Cultural reservations; Gender-related reservations; Emotional reservations; Privacy preservation; Defensiveness and negative reactions; Other: type of treatment.	positive experience of the situation; Other: appearance	Information and being informed; consent to participate; confidentiality - privacy - data protection; Other: role of students.	
Alawad and Younis [13]	Patients of a university hospital in Sudan	432, age 40.7 average, 43.7% female	Acceptance; personal benefits, altruism	Gender-related reservations; Emotional reservations; Other: presence of teacher, type of treatment, level of education.	positive experience of the situation; language of those involved	Information and being informed; consent to participation	Group size
Aljoudi et al. [14]	Adult patients at a university hospital in western Saudi Arabia.	367 patients	Acceptance	Gender-based reservations; privacy preservation; defensiveness and negative reactions; Other: type of treatment, level of training, type of student dress, student behavior and skills.	positive experience of the situation	Information	
Anfinan et al. [15]	Inpatients and outpatients of a university hospital in Saudi Arabia	327 patients, age 17–83 years	Acceptance; Own experiences; altruism.	Cultural reservations; Religious reservations; Gender-related reservations; Other: appearance, behavior, dress, manner of treatment.	positive experience of the situation; re-participation	Consent to participation	Group size
Arolker et al. [16]	Inpatients and outpatients of a hospice who have been involved in teaching medical students	15 patients and 14 employees	Acceptance; personal advantages (benefit); altruism		positive experience of the situation; language of the participants; Other: role as teacher, confrontation with own illness	Consent to participation	
Ben Salah et al. [17]	Patients from 22 departments of a university hospital. For participants in pediatrics under 15 years of age, parents were interviewed.	356 patients, including 30 under 6 years of age and 317 adults	Acceptance; personal benefits, distraction from everyday life; systemic constraints; altruism. Other: improving quality of health care.	Cultural reservations; Religious reservations; Gender-related reservations; Emotional reservations; Privacy preservation; Defensiveness and negative reactions; Other: type of treatment, age, low confidence.	positive experience of the situation; negative experience of the situation; Other: feelings of shame	Information and being informed; consent to participate; confidentiality - privacy - data protection.	Premises
Bukhari et al. [18]	Outpatient dermatology patients in contact with medical students.	102 patients	Acceptance; personal advantages (benefit)	Cultural reservations; Gender-related reservations; Privacy preservation; Other: age	positive experience of the situation; re-participation	Confidentiality - privacy - data protection	
Callaly et al. [19]	Patients admitted as inpatients, age 18–58 years	92	Altruism	Cultural reservations; Religious reservations; Gender-related reservations	Positive experience of the situation; participation; language of those involved; Other: interruptions (meals, visit), trust, positive relationship with the health professionals	Information and being informed; confidentiality - privacy - data protection	Duration; location
Carey et al. [4]	Women, age 18–70 years	401	Acceptance; personal benefits; systemic constraints; altruism); Other: educational support	Gender reservations; Emotional reservations; Privacy preservation; Other: overwhelm, discomfort, shyness.	positive experience of the situation; effort and fatigue; language of the participants	Information and being informed; consent to participate; confidentiality - privacy - data protection; Other: desire for verbal and written information.	location
Carty et al. [20]	Women, age 24–80 years	22 patients	Acceptance; personal benefits; systemic constraints; altruism	Emotional reservations; defensiveness and negative reactions.	positive experience of the situation; effort and fatigue; perceptions of safety; participation; language of participants; Other: students are seen as collaborators	Information and being informed; consent to participate; confidentiality - privacy - data protection; perspective of guardians.	Group size; location
Ching et al. [21]	Patients of gynecological health centers of a university hospital with contact with medical students	180 patients	Acceptance; own experiences; personal benefits; altruism	Cultural reservations; gender-related reservations; privacy preservation; defensiveness and negative reactions; other: level of training, type of treatment, comfort of treatment.		Confidentiality - privacy - data protection	
Choudhury et al. [22]	Patients from two hospital outpatient clinics and two general medical practices in London	422 patients	Acceptance; own experiences; altruism; other: importance of participation for medical student education.	Cultural reservations; Gender-related reservations; Emotional reservations; Defensiveness and negative reactions; Other: type of treatment, presence of teacher, health status, ethnicity.	positive experience of the situation	Information and be informed	Group size
Chretien et al. [23]	Inpatients in acute care who had participated in a physical examination during a teaching session for third-year medical students, predominantly men aged 52–79 years	12	Acceptance; own experiences; personal advantages, distraction from everyday life; systemic constraints; altruism; other: learn something yourself	Preservation of privacy; defensiveness and negative reactions; Other: unable in health, interruptions to meal times.	positive experience of the situation; negative experience of the situation; participation; language of the participants; Other: smile and good communication	Information and being informed; Other: information about bedside teaching could improve participation.	Group size; location; Other: process flow
Figueiró-Filho et al. [24]	Pregnant women over the age of 18, medical students in their fifth or sixth year of training.	95 female patients, 81 students	Acceptance; Own experience	Emotional reservations; Other: reservations about treatment, lack of trust.	positive experience of the situation; feelings of safety; other: treatment success, anxiety	Consent to participation	Other: teaching method (hardly guided)
Franks and Rudd [25]	Patients of an inpatient hospice	22	Personal benefits; distraction from everyday life; altruism; Other: appropriate time	Other: concern of the nurses	positive experience of the situation; perceptions of safety; participation; language of participants	Information and being informed; consent to participate; confidentiality - privacy - data protection.	
Gebrekirkos and Van Wynk [26]	Adult patients (61% male)	127		Privacy preservation; defensiveness and negative reactions; Other: trust, educational attainment.	Positive experience of the situation; negative experience of the situation; perceptions of safety; language of the participants; Other: no knowledge of the presence of the students	Information and being informed; consent to participate; confidentiality - privacy - data protection.	
Al Ghobain et al. [27]	Adult patients from medical and surgical wards of a university hospital in Saudi Arabia, age 18–107 years.	416	Acceptance; Altruism	Gender reservations; privacy preservation; other: teacher presence.	positive experience of the situation; negative experience of the situation; perceptions of safety; re-participation; Other: educational benefit	Information and being informed; consent to participate; confidentiality - privacy - data protection.	
Gillon et al. [28]	inpatient hospice patients	122	Acceptance		positive experience of the situation		Group size
Harris et al. [29]	Published work examining the views of terminally ill patients on participation in undergraduate medical education.	7 articles, with a total of 269 patients	Acceptance; personal benefits; distraction from everyday life; systemic constraints; altruism	Emotional reservations; preservation of privacy; defensiveness and negative reactions; other: fear of treatment failure.	positive experience of the situation; effort and fatigue; perceptions of safety; participation; re-participation.	Consent to participation	
Hartz and Beal [30]	Patients of two clinics for gynecology and obstetrics, mean age 34.9 years	213	Acceptance; personal benefits; altruism	Gender-based reservations; preservation of intimacy; preservation of privacy; Other: presence of teacher, type of treatment, level of education.	Perceptions of safety; language used by stakeholders	Information and being informed; confidentiality - privacy - data protection	Group size
Iqbal et al. [31]	Outpatients and inpatients of a provincial hospital without acute treatment needs. Average age 36.8 years, 64.7% female	196 patients contacted, 187 consenting participants		Cultural reservations; gender-related reservations; privacy preservation; defensiveness and negative reactions; other: reservations about treatments, married persons.	positive experience of the situation	Information and being informed; consent to participate; confidentiality - privacy - data protection; perspective of guardians.	
Khoo et al. [32]	Parents who participated with their children in bedside teaching of medical students; 83% were mothers with an average age of 32.1 years (19–50 years)	54	Acceptance; own experiences; personal advantages; altruism; other: cooperation between parents and medical staff	Cultural reservations; Religious reservations; Emotional reservations; Privacy preservation; Other: Child welfare risk, Health impairment.	positive experience of the situation; negative experience of the situation; effort and fatigue; participation; language of the participants	Information and being informed; consent to participation; view of instructors; Other: children’s voluntariness.	Group size
Kianmehr et al. [33]	Medical students, patients over 18 years of age with a length of stay of more than 48 h at a university hospital	100 students and 100 patients	Acceptance; personal benefits; distraction from everyday life.	Religious reservations; Emotional reservations; Privacy preservation; Defensiveness and negative reactions.	positive experience of the situation; participation; language of the participants; Other: improvement of communication between doctor and patient	Information and being informed; confidentiality - privacy - data protection	
Klaber and Pollock [34]	26 children and teenagers (10 to 17 years), 54 parents,	80 of 112 questionnaires completed	Acceptance; own experiences; personal advantages, systemic constraints; altruism; other: voluntary, financial incentive, parents decide on participation	Emotional reservations; defensiveness and negative reactions; Other: boredom, aversion to the hospital atmosphere, fear of treatment errors or rough treatment.	positive experience of the situation; negative experience of the situation; effort and fatigue; participation; re-participation; Other: rigor, joy.	Information and being informed; consent to participate; confidentiality - privacy - data protection; perspective of instructors; Other: presence of parents.	Duration; Other: Number of examinations
Koehler and McMenamin [35]	Publicly sought participants 18 years and older (male = 134; female = 268), average age 27.7 (18–76).	402 answered at least one question, 339 answered the entire questionnaire	Own experience	Cultural reservations; Religious reservations; Gender-related reservations; Emotional reservations; Intimacy preservation; Privacy preservation; Defensiveness and negative reactions; Other: type of treatments, level of student training, treatment errors.	positive experience of the situation; re-participation	Information and being informed; confidentiality - privacy - data protection	Group size
Kriesen et al. [36]	Patients, medical students and staff of a palliative care department of a university hospital.	20 patients, 21 students and 19 employees	Acceptance; personal benefits; distraction from everyday life; altruism	Emotional reservations; privacy preservation; Other: health impairment, symptom burden.	positive experience of the situation; negative experience of the situation; effort and fatigue; feelings of safety; Other: reflection of own negative situation	Information and being informed; consent to participate; Other: possibility of interruption.	Group size; duration; location; Other: slow process.
Kuan and O’Donnell [37]	Outpatients over 16 years of age of an emergency department	Response 145 patients, 45 (31%) of whom were in contact with medical students.	Acceptance; own experience; systemic constraints.	Emotional reservations; preservation of privacy; defensiveness and negative reactions; Other: fear of mishandling, No trust, type of treatment, feeling pushed.	Positive experience of the situation; negative experience of the situation; perceptions of safety; Other: prolongation of treatment time, lack of guidance.	Information and being informed; consent to participation	Duration; Location
Khan et al. [38]	Patients of the surgery department of a university hospital	411 patients, of whom 279 were in contact with students and 132 were not.	Acceptance; own experiences; altruism	Preservation of privacy; defensiveness and negative reactions; Other: longer waiting time, guidance is disruptive, type of treatment.	positive experience of the situation; negative experience of the situation; Other: instruction is experienced as disturbing	Consent to participation	Group size
Law et al. [39]	Patients over 18 years of age of a rural general hospital.	45 patients (86% female), of whom 18 had no contact with students and 27 had contact with students	Acceptance; personal benefits; systemic constraints; altruism; Other: disease understanding is higher.	Emotional reservations; preservation of privacy; defensiveness and negative reactions; Other: type of treatment.	positive experience of the situation; negative experience of the situation; re-participation	Information and being informed; consent to participate; confidentiality - privacy - data protection.	Other: Type of supervision
Lucas and Pearson [40]	Patients who voluntarily participated in an arranged learning session	18	Acceptance; own experiences; personal benefits; distraction from everyday life; altruism		Positive experience of the situation; negative experience of the situation; effort and fatigue; perceptions of safety; participation; language of participants; Other: respect for the patient, insight into medical training, gratitude.	Information and being informed; consent to participation	Duration
Lucas et al. [41]	Patients of a trauma surgery ward of a university hospital	162	Acceptance; altruism; other: type of treatment, satisfaction with physician relationship	Emotional reservations; privacy preservation; privacy preservation; Other: number of learners	positive experience of the situation; language of those involved		Group size; location
Marwan et al. [42]	Patients of different primary, secondary and tertiary care medical units in Kuwait.	932 patients, age 0–89, 53.4% female, 94% Muslims	Acceptance; personal advantages; other: education, discipline	Cultural reservations; Religious reservations; Gender-related reservations; Privacy preservation; Defensiveness and negative reactions; Other: type of treatment, presence of teaching staff, teaching affects quality of treatment, level of training	positive experience of the situation; feelings of safety; participation	Consent to participation; confidentiality - privacy - data protection; perspective of guardians.	Duration; location
McKimm [43]	---	---	Acceptance; own experiences; personal benefits; distraction from everyday life; systemic constraints; altruism	Cultural reservations; Religious reservations; Gender-related reservations; Emotional reservations; Privacy preservation; Defensiveness and negative reactions; Other: level of education, type of treatment, health status.	Perceptions of safety; Participation; Other: role of the teacher, feedback from patients.	Information and being informed; consent to participate; confidentiality - privacy - data protection.	Group size; duration; location
Monnickendam et al. [44]	Patients over 18 years of age in family medicine practices.	375	Acceptance; systematic constraints	Gender-based reservations; privacy preservation; privacy preservation	positive experience of the situation; feelings of security; Other: type of treatment, presence of the teacher	Information and being informed; consent to participation	
Mora-Pinzon et al. [45]	Patients of a surgical department of a university hospital, (57% female, age 26–86).	84 patients	Acceptance; systemic constraints; other: no objections	Defensiveness and negative reactions; Other: disease state	Positive experience of the situation; language of those involved; Other: better care and treatment, more time spent, more active listeners.	Consent to participation	Duration; location; Other: Time of bedside teaching
Mosalanejad et al. [2]	Patients of a university hospital who were present at bedside teaching, majority from the age group 51 to 60 years old	400	Acceptance; personal advantages		positive experience of the situation; participation; language of the participants; Other: more knowledge about own disease and treatment, possibility to pass on knowledge to students and teachers		
Nair et al. [46]	Patients, students, interns and residents of a university hospital.	160 patients, 78 students, 58 interns, 64 residents	Acceptance	Emotional reservations; preservation of privacy; preservation of privacy.	positive experience of the situation; negative experience of the situation; perceptions of safety; participation; language of participants; re-participation.	Information and be informed	location
O’Flynn et al. [47]	Patients of teaching physicians, 60% female, average age 50 years.	335 patients	Acceptance; own experience; systemic constraints; other: clinical situational dependence.	Gender-based reservations; Emotional reservations; Privacy preservation; Defensiveness and negative reactions; Other: type of treatment.	negative experience of the situation; re-participation	Information and being informed; consent to participate; confidentiality - privacy - data protection.	location
Onotai et al. [48]	Adult patients at a university hospital in Nigeria, average age 39.3 years.	240	Acceptance; personal advantages	Religious reservations; Gender-related reservations; Privacy preservation; Other: age,	positive experience of the situation; negative experience of the situation; Other: type of treatment	Information and being informed; consent to participation	Group size
Petersen et al. [49]	Patients of an ambulatory and stationary general medical care	254, of which 120 in the examination room and 134 in the conference room	Personal advantages	Emotional reservations; Other: Time of treatment.	positive experience of the situation; participation; language of the participants; other: reduced autonomy	Information and being informed; confidentiality - privacy - data protection	Duration; location; Other: degree of training, compliance.
Rizk et al. [50]	Women of a university department of obstetrics and gynecology	264	Acceptance; own experiences; personal benefits; systemic constraints; altruism	Cultural reservations; Religious reservations; Gender-related reservations; Privacy preservation; Other: presence of teacher, type of treatment.	Positive experience of the situation; negative experience of the situation; participation; Other: ambiguity about the role of students.	Information and being informed; consent to participate; confidentiality - privacy - data protection.	
Rockey et al. [51]	Patients 18 years of age or older at a university hospital who participated in a teaching unit taught by outside instructors, (ages 19–93)	111 patients	Acceptance; own experiences; personal benefits; distraction from everyday life; systemic constraints; altruism; other: teaching as a task of patients.	Emotional reservations; defensiveness and negative reactions; Other: preserving autonomy, not knowing what to expect, pain.	positive experience of the situation; negative experience of the situation; effort and fatigue; feelings of safety; participation; language of participants; re-participation; Other: pain, process takes too long, have received further knowledge about own illness, treated with respect, have had to experience conversations about oneself, variety, have had to repeat own illness story	Information and being informed; consent to participate; confidentiality - privacy - data protection; Other: language comprehension.	Duration
Rubliauskas et al. [52]	Patients of the surgical department of a university hospital	150	Acceptance; personal benefits; altruism	Emotional reservations; defensiveness and negative reactions; Other: lack of professionalism of students, nature of treatment	Participation; Other: quality of treatment	Information and be informed	Group size
Sayed-Hassan et al. [53]	Patients from three university hospitals (mean age 40.2 years, 66% female).	400	Acceptance; personal benefits; systemic constraints; altruism; other: higher education level, had no other choice,	Cultural reservations; Religious reservations; Gender-related reservations; Emotional reservations; Preservation of privacy; Defensiveness and negative reactions; Other: participation of teachers, type of treatment.	positive experience of the situation; feelings of safety	Information and being informed; consent to participate; confidentiality - privacy - data protection.	Group size; location
Shetty et al. [54]	Patients from different departments of a hospital (34% female)	200 patients	Acceptance; own experiences; personal advantages, altruism; other: higher educational qualifications	Cultural reservations; Gender-related reservations; Emotional reservations; Privacy preservation; defensiveness and negative reactions; other: type of treatment, presence of a teacher, stress, health concerns.	positive experience of the situation; language of those involved; Other: medical staff hierarchy, student dress, language barriers, talking about the patient,	Information and being informed; consent to participate; confidentiality - privacy - data protection; perspective of guardians.	Group size

### Patient motivations for participation

Patient willingness to accept student involvement varied considerably across studies, although it was generally positive (see Table 3).

**Table 3:** Acceptance and satisfaction

Study ID	Acceptance and satisfaction
Abdulghani et al. [12]	Do you cooperate with medical students as you cooperate with your physician? (*n* = 474):16,4% yes, 83.6% no
Alawad and Younis [13]	Do you approve the existence of medical students during consultation? (*n* = 432): 95.2% yes, 4.8% no
Aljoudi et al. [14]	I would be comfortable to have a medical student involved by observing the doctor asking me questions (*n* = 367): 4.01 ± 0.91 overall comfort score
Anfinan et al. [15]	179 participants (54.7%) reported a positive attitude regarding the presence of female medical students and 40.3% (*n* = 132) reported positive attitudes regarding the presence of male medical students during consultations.
Arolker et al. [16]	Qualitative interviews (*n* = 15): “Patients commonly stated that they could not think of a reason why any patient would not want to participate, a perspective that illustrates patient positivity toward the teaching encounter.”
Ben Salah et al. [17]	Level of satisfaction by the care provided by medial students (*n* = 356): 39% very satisfied; 54.2% satisfied; 6.8% not satisfied or not satisfied at all
Bukhari et al. [18]	Enjoyed interacting (*n* = 102): Strongly agree 41.2%, agree 27.5%, undecided 16.7%, disagree 3.9%, strongly disagree 10.8%.
Callaly et al. [19]	Did you enjoy talking to the medical student? (no = 0 to yes = 10) (*n* = 92): 9.13 ± 1.16
Carey et al. [4]	Taking part in BST could be an enjoyable experience for me (*n* = 401): 20.4% strongly agree; 38.2% agree; 28.7% neutral; 8.5% disagree or strongly disagree (4.2% does not apply or not Response)
Carty et al. [20]	Qualitative interviews (*n* = 22): 15 statements about positive experiences
Ching et al. [21]	My comfort level with having the examination procedure done by a medical student. Acceptance: 44% (*n* = 66)
Choudhury et al. [22]	Would you allow medical students to be present? Yes 91.95% (*n* = 377), no 8.05% (*n* = 33)
Chretien et al. [23]	Qualitative interviews (*n* = 12): “Participants hold a positive impression of medical student education”
Figueiró-Filho et al. [24]	Comfort with student consultation (*n* = 95): Before encounter: 77.9% Agreement; 12.6% Disagreement; 9.5% No opinion. After encounter: 96.8% Agreement; 1.1% Disagreement; 2.1% No opinion
Franks and Rudd [25]	Did you enjoy having them [the students] come to see you? 91% (*n* = 20) enjoyed it, 9% (*n* = 1) neutral
Gebrekirkos and Van Wynk [26]	I like it when there are students around me: strongly agree (*n* = 3), agree (*n* = 15), never thought about it (*n* = 4), disagree (*n* = 101), strongly disagree (*n* = 4)
Al Ghobain et al. [27]	I accept the participation of medial students in my healthcare (*n* = 416): agree 88%, 12% disagree
Gillon et al. [28]	The majority of participants were happy for students to be present on the ward round (*n* = 106) 84%.
Harris et al. [29]	Systematic Review: Most patients were happy that they had agreed to take part in medical student teaching (21/22 patients, 95%). Participating in teaching was “very” or “quite” enjoyable (27/27 patients, 100%).
Hartz and Beal [30]	“A majority of patients reported they would feel comfortable having a medical student present during most clinical situations [.]”.
Iqbal et al. [31]	Would you permit medical students to be present in the outpatient clinic if you were having a consultation with your doctor? (*n* = 187): males only 0.5%, females only 11.8%, both 81.3%, neither 6.4%
Khoo et al. [32]	Qualitative interviews (n = 54): " In general, parents viewed BST sessions involving their children positively".
Kianmehr et al. [33]	Do you satisfied with BST? (*n* = 100): 60% Yes; 40% No
Klaber and Pollock [34]	Enjoy time with students and doctors: children (*n* = 26) 27% strongly agree; 35% agree; 38% neither; 0% disagree or strongly disagree; parents (*n* = 54) 15% strongly agree; 34% agree; 38% neither; 11% disagree; 2% strongly disagree
Koehler and McMenamin [35]	“In general, the further the body parts were away from intimate body regions (e.g. genitals), the more likely individuals were to permit less advanced medical students to examine them.”
Kriesen et al. [36]	Qualitative interviews (*n* = 20): What were your experiences in regard to bedside teaching? - “Patients in palliative care situations were previously cited to consider bedside teaching as valuable (Frank et al. 1997). Indeed, the responses of our patients taking part in the present study support this view.”
Kuan and O’Donnell [37]	“Of those with previous student contact, 35 (78%) had positive experiences, 4 (9%) negative and 6 (13%) were unsure” (*n* = 145)
Khan et al. [38]	Patients’ receptiveness of their own involvement in student teaching (*n* = 411):41% strongly agree; 29% agree; 6% neutral; 16% disagree; 8% strongly agree
Law et al. [39]	Satisfaction ratings of patients seen by a physician with (*n* = 27) or without a medical student (*n* = 18) present: with students 43.07 out of 45/without students 42.72 out of 45
Lucas and Pearson [40]	Qualitative interviews (*n* = 18): “All participants were satisfied with the organization of patient teaching including both the preparation and process of their involvement.”
Lucas et al. [41]	Motivation to participate in bedside teaching (*n* = 162): “Overall motivation to participate in UaK was found to be high (3.97)”
Marwan et al. [42]	Would you permit medical students to be present in the ward rounds if you were admitted in the same ward? (*n* = 932): 3,8% only males; 9,4 only females; 77,3% both males and females; 9.5% neither males nor females
McKimm [43]	General evidence of patient satisfaction: “Most research into patient views on involvement in teaching emphasizes the positive nature of the encounter, ‘even unprepared patients see themselves as contributors to teaching’ (Haffling and Håkasson 2008).”
Monnickendam et al. [44]	I am happy for a student to be present during my consultation (*n* = 375): disagree/strongly disagree 3.2%, did not answer 2.1%, agree or strongly agree 76.8%
Mora-Pinzon et al. [45]	Overall patient’s attitude toward medical students (*n* = 82): 44% No objections to medical students; 47.6% preferred a limited involvement; 8,3% refused medical students
Mosalanejad et al. [2]	“59.9% of the patients evaluated the bedside teaching as high and very high, 26.5% average, and only 3.3% low and very low.” (*n* = 400)
Nair et al. [46]	Did you enjoy it (*n* = 100): 77% yes; 17% no
O’Flynn et al. [47]	“In both samples, 95% of patients (141/149, London; 176/186, Newcastle) were happy for students to be present during an examination again”
Onotai et al. [48]	“One hundred and eighty-five (77.1%) of respondents indicated their willingness to allow students participate in their care, while 55 (22.9%) of respondents indicated their unwillingness to allow student participation […].”
Petersen et al. [49]	Overall satisfaction with visit: exam room 4.4 ± 1.0 (*n* = 120), conference room 4.5 ± 1.0 (*n* = 134)
Rockey et al. [51]	I felt comfortable with being asked to participate in medical student physical exam/history taking teaching. (*n* = 110): 89% strongly agree; 18% agree; 1% neutral; 2% disagree or strongly disagree
Rubliauskas et al. [52]	Would you permit medical students to be present during your surgery one more time? (*n* = 150): 78% yes; 22% no
Sayed-Hassan et al. [53]	Do you approve the existence of medical students during clinical encounter? (*n* = 400): 67.8% yes; 14.8% no; 17.3% does not matter
Shetty et al. [54]	Patients’ views on the involvement of students in general examination (*n* = 200): 98% students allowed; 1% No students allowed, because of confidentiality; 1% No students allowed, because of health concerns.

The number of patients who indicated that they generally refused to participate in practice guidance varied. Most patients across studies indicated a willingness to accept student involvement in their care, with acceptance rates ranging from 78% to 100%: 78% (*n* = 27) [29], 100% (*n* = 80) [34], 93% (*n* = 27) [39], and 78% (*n* = 117) [52]. However, acceptance varied substantially by procedure type and context. For instance, a Saudi Arabian study revealed that 43.3% of 196 respondents refused to allow students to perform investigative procedures [31], while some patients declined participation even when the educational importance was acknowledged [35].

Patient motivations for accepting student involvement are clustered into two overarching categories: personal benefits and altruism. Personal benefit motivations (reported in 15 studies [2, 4, 13, 16, 18, 19, 21, 23, 29, 30, 40, 42, 43, 48, 50] included a) improved understanding of their own condition through extended explanations, b) increased time and attention from health care staff, and c) perceived higher quality of care through additional oversight. Altruistic motivations (reported in 8 studies [13, 16, 19–21, 25, 29, 43]) centred on helping students develop professional competencies, contributing lived experience to education, and “giving back” to the health care system. Additionally, some patients described themselves as active partners or co-educators in the teaching process [20].

The respondents cited personal benefits, such as being able to learn more about their own disease, as a reason for their participation in medical student learning units [2, 4, 13, 23, 30, 42, 43, 48, 50]. Patients feel that physicians spend more time with them when students are present and perceive these instances as higher-quality care [18, 21, 42, 45, 48]. Hospitalized patients’ participation in practice guidance sometimes provides a welcome break from daily routines and opportunities for social interaction [23, 29, 33].

Not all individuals perceive participation in teaching units as beneficial; some participate reluctantly or because it is expected of them in the situation [23]. Not having a choice or not being able to “say no” [43, 44, 47] is also noted in some studies as a reason for participation.

### Barriers and reasons for refusal

 Privacy concerns, particularly related to intimate examinations, represented the most frequently cited barrier to student participation. Examinations or procedures involving the genital area were most often declined by patients [12, 21, 35]. The desire for privacy and dignity in sensitive clinical situations—including childbirth, gynaecological care, and mental or sexual health consultations—was consistently emphasized across studies [14, 18, 20, 21, 23, 30, 35, 36, 38, 43, 54]. Some patients declined any observers during such procedures [35], whereas others refused to discuss sexual or personal topics in the presence of students [12, 42, 51].

 Gender concordance preferences were particularly pronounced in obstetric and gynaecological contexts. Women were significantly more likely to decline allowing the participation of male students in gynaecological examinations and obstetric care [12–14, 17, 31, 35, 42, 50, 54]. Shetty et al. [54] characterized this response as “same-sex preference,” a phenomenon also observed, although less frequently, among male patients [18, 27]. Notably, despite these preferences, 81.1% (*n* = 95) of women acknowledged that students require practical experience in gynaecology and obstetrics [24], suggesting tension between personal comfort and educational awareness.

Patient confidence in student competence emerged as another significant barrier. Patients found it challenging to assess the risks associated with student-performed examinations and to evaluate student skill levels [20, 31, 35, 52]. Students’ training level influenced acceptance rates, with more advanced students receiving greater acceptance [14, 35]. Patients generally expected students to self-assess their abilities accurately and respect their limitations [36]. However, only a minority of patients reported that student involvement negatively affected their health care quality [28, 29, 52].

 Anxiety was commonly reported among patients prior to clinical teaching participation [24, 26]. In one study of pregnant women, 22% (*n* = 95) expressed initial uncertainty about trusting students; however, following the teaching encounters, 97% reported positive experiences [24]. Patient anxieties stemmed from multiple sources: concerns about procedural pain [36], reluctance to be reminded of their illness [43], and anticipated stress from the teaching situation [54]. These findings suggest that pre-participation anxiety does not necessarily predict negative experiences, highlighting the importance of preparation and communication.

The influence of cultural and religious factors on student acceptance has yielded inconsistent findings across studies. Anfinan et al. [15] identified religious beliefs as the primary reason for declining student presence during rounds and consultations in their study population (*n* = 138; 42.2%). However, another study conducted in a predominantly Muslim country revealed no clear relationship between religion and participation willingness [42]. Similarly, studies from the U.S [21]., Ireland [19], and Australia [35] did not identify significant relationships between cultural background and acceptance rates. These mixed findings suggest that cultural and religious attitudes do not universally determine participation decisions. Individual preferences, procedural context, communication quality, and institutional practices may be more influential than cultural or religious affiliation per se.

### Patient evaluations of clinical education experiences

Patients generally reported positive experiences with student involvement in their care, with high levels of patient satisfaction (see Table 3). However, satisfaction levels varied depending on multiple contextual and procedural factors.

Beyond passive acceptance, some patients described having meaningful engagement with the educational process. Forms of patient participation ranged from providing case documentation to active involvement in clinical demonstrations [40, 43]. Some patients reported that participation in bedside teaching enhanced their sense of involvement in their own treatment process [2], while students and educators gained valuable insights from patient perspectives [2, 33, 43]. This reciprocal dynamic suggests the potential for conceptualizing patients as partners in clinical education rather than merely as passive subjects.

#### Negative experience

However, the experiences were not uniformly positive. Some patients reported negative experiences, including physical discomfort during prolonged examinations, boredom with extended educational discussions, and psychological discomfort when information about the student roles was inadequate. Boredom emerged as a concern for specific patient populations, particularly those with terminal illnesses [25, 29, 51] and paediatric patients [34]. Similarly, some parents reported that their children found extended teaching sessions tiring [32]. These findings suggest that the duration and structure of teaching encounters require careful consideration, especially for vulnerable populations.

#### Physical discomfort

In a qualitative survey of palliative care patients, Kriesen et al. [36] reported that symptom distress among participants was a reason for dropping out of bedside teaching. In a survey by Rockey et al. [51], 48% (*n* = 109) agreed that they experienced mild-to-severe pain during participation. Some patients complained that they were not treated carefully or were treated roughly during the examinations [34, 51].

A subsequent assessment is influenced by students’ lack of experience or inadequate guidance from instructors [37, 43]. The presence of instructors and good guidance can be beneficial for reducing patients’ fears in sensitive situations and for creating a sense of security [29]. Conversely, inadequate supervision or limited student experience contributed to negative patient assessments.

In this context, the forms of patient participation in practical training range from providing documentation as case studies to active participation in examinations [41, 43]. Participation in bedside teaching may thus also lead to greater perceived patient involvement in the treatment process [2]. Students and educators can also benefit from further information provided by patients [2, 43, 48].

### Information provision and consent processes

Communication quality and language accessibility emerged as significant determinants of positive patient experiences. Patients expressed a preference for explanations that avoided or clarified medical terminology [4, 32], facilitating a better understanding of their own conditions [31]. The tone and manner of communication also influenced patients’ sense of autonomy, with respectful, collaborative communication styles enhancing self-determination, while directive or dismissive approaches engendered feelings of powerlessness [20].

In multilingual contexts, language concordance was identified as beneficial. When students could communicate in patients’ preferred languages [54] or when teaching discussions were translated for patients [26], their satisfaction and comfort levels increased.

#### Preliminary information for patients and patients’ rights

Adequate information provision and informed consent constitute fundamental ethical requirements for patient participation in clinical education. However, research has identified substantial gaps between ethical ideals and actual practice. Patients expect to receive verbal or written information in advance [4, 12, 13, 33, 44, 54]. This information includes the facility’s role as a teaching institution and its involvement in professional education [47], their role as patients [24, 43] and whether they are being treated by a student [17, 42, 43, 47].

Despite these expectations, information provision was frequently inadequate. Patients did not always receive information or failed to recall receiving it [26, 46]. In a survey by Kuan and O’Donnell [37], only one of 145 participants recalled reading information about student involvement. Similarly, Shetty et al. [54] reported that approximately two-thirds of respondents were unaware of the institutional hierarchy (76%, *n* = 152) and role distribution (74.5%, *n* = 149) within the clinical team.

When adequate information was provided, studies reported improved outcomes [18, 23], greater acceptance of participation, and reduced patient anxiety [22]. These findings demonstrate that information provision is not only a bureaucratic formality but also a substantive contributor to patient experience quality and ethical practice.

#### Privacy and confidentiality concerns

Privacy and confidentiality concerns represented another barrier to comfortable participation. Patients sometimes expressed that when students were involved, their privacy was not respected and that their records were not handled confidentially [12–14, 46, 48, 50]. Carty et al. [20] reported that the ability of teaching participants to ensure patient information confidentiality is critically important for maintaining patient trust and ethical standards. The voluntary nature of patient consent emerged as a critical ethical concern. While only a minority of patients declined participation, obtaining genuine prior consent was emphasized as essential regardless of acceptance rates [25, 29, 30].

However, the consent processes were sometimes problematic. Patients reported feeling coerced to agree to participate [13, 29]. In Rockey et al.’s [51] study, 52% (*n* = 109) of respondents felt that they were expected to participate, suggesting perceived pressure rather than an authentic choice. This finding raises questions about the meaningfulness of consent when institutional cultures implicitly pressure patients for their acceptance. Conversely, studies explicitly emphasising on voluntary participation and clear communication on the right to refusal have shown that patients felt respected and valued [16, 20]. These findings suggest that consent quality depends not only on formal procedures but also on the institutional culture and the manner in which the choice is presented and honoured.

#### Needs of children and parents

Children and their parents have special needs as participants in practice guidance, which has been specifically considered during previous studies. Khoo et al. [32] emphasized the importance of obtaining both parental consent and, when developmentally appropriate, child assent—recognizing children as persons with emerging autonomy rather than merely as subjects of parental decision-making. Parental presence during teaching activities with young children was considered essential by some parents [20], reflecting both their protective instincts and recognition of the children’s need for familiar support in clinical settings. Many parents believed that participation in teaching situations could facilitate connections between clinicians, students, and their children [32], suggesting the perception of potential relational benefits. However, parents also emphasized the importance of their involvement in planning teaching activities to minimize potential stress and anxiety for their children [33]. This finding highlights the need for developmentally appropriate approaches that recognize children’s vulnerability while respecting families’ preferences and expertise regarding their children’s needs. Interestingly, refusal rates in paediatric settings varied across studies, with Iqbal et al. [31] reporting low refusal rates and Marwan et al. [42] reporting higher acceptance rates.

### Educational and organizational context factors

Research has examined how organizational aspects of clinical education affect patient experiences, including teaching location, group size, and timing. Patient preferences for the teaching location and modality varied depending on the nature of the clinical activities and the individual circumstances. The differences between bedside teaching [46] in examination and meeting rooms [49] and digital consultations have been analysed [4].

Student group size was further investigated as a factor influencing patient satisfaction. Small groups were generally preferred, with four or fewer learners representing a common maximum threshold that patients found acceptable [22, 28, 53, 54]. However, Carty et al. [20] and Harris et al. [29] reported that context-specific variations preclude universal recommendations for optimal group size, as acceptability depends on the clinical situation, procedure type, and individual patient preferences. Large groups were associated with patient discomfort [35, 48], particularly in contexts involving intimate examinations, sensitive conversations, or vulnerable patient populations.

The temporal organization of teaching activities significantly influenced patient experiences. Patients identified multiple problematic patterns: they identified concerns about educational activities that were perceived as excessively long or repetitive, poorly timed (during meals or late in the day) or that resulted in increased waiting times for care [19, 29, 38, 48, 51]. The repetition of summaries or examinations by students [42] and the ability to answer student questions or participate in multiple teaching sessions [20, 34] are sometimes considered wastes of time by patients.

## Discussion

This scoping review synthesized evidence from 45 papers involving 9,538 patients to examine patient perspectives on student participation in clinical teaching. Studies that exclusively examine specific instructional situations in nursing are lacking.

Patient acceptance of student involvement was generally high, with most studies reporting acceptance rates exceeding 75%. Despite the high acceptance rates, important barriers emerged. Privacy concerns—particularly regarding intimate examinations—represented the most consistent barrier across studies. Competence-related anxieties, inadequate information provision, problematic consent processes, and organizational factors (large student groups, poor timing, and excessive duration) also negatively influenced patient experiences. The tension between high reported acceptance rates and evidence of perceived coercion warrants critical examination. When more than half of the participants in some studies felt “expected” to participate, the meaningfulness of consent became questionable. This observation suggests that reported acceptance rates may partially reflect institutional power dynamics and a perceived inability to refuse rather than authentic choice. Health care institutions must distinguish between formal consent compliance and genuine voluntariness.

### Limitations

The evidence found in these studies was limited, and comparability was not provided. In the six qualitative interviews, 12 to 54 people were surveyed on various considerations. In the 37 quantitative studies and interviews, neither a uniform questionnaire nor comparable sociodemographic characteristics were collected.

No study has provided a comparable overview of survey participants’ sociodemographic data, such as age, sex, diagnosis, or residency status [29]. The number of respondents was small (≤ 500) [29]. Therefore, patient feedback on participation in health professional education should be standardized to obtain more comprehensive and valid data. The search strategy, while systematic, may not have captured all the relevant literature, particularly from non-English-language sources or practice-oriented publications. No quality assessment of the included studies was conducted, which is consistent with the scoping review methodology, but the ability to weigh evidence by rigor is limited.

### Implications for nursing education

A fundamental limitation of this review is that all the identified evidence is derived from medical education contexts. While this scoping review was explicitly designed to examine medical education evidence for its potential applicability to nursing, the absence of nursing-specific research represents a critical gap that requires careful consideration when interpreting implications. While medicine and nursing share patient-centred values, nursing education involves distinct elements: sustained patient interaction, intimate personal care activities (versus examinations), closer relational dynamics, and greater family involvement. These differences mean that medical education findings cannot be directly extrapolated without adaptation. Additionally, no nursing-specific research was identified for outpatient, long-term care, or community settings, limiting generalizability. The absence of nursing-specific evidence across diverse contexts and care activities represents a critical gap that requires urgent research attention to ensure that the recommendations are contextually appropriate and ethically sound.

Despite these substantial limitations, the review identifies several core ethical principles that appear to be foundational across health profession education.

Institutions should implement structured informed consent processes to adapt to nursing contexts, in which sustained intimate care activities differ from brief medical examinations.

### Recommendations for practice

The reviewed studies described a high level of acceptance of participation in medical education. Furthermore, most patients were satisfied with the process and their participation. The following recommendations can be derived from this review:

- It is important to consider patients' rejection or wishes, especially because of their dependency on care [40].
- Obtaining permission and informed consent are mandatory [26, 32, 55, 56].
- Patients must be actively informed of the privacy and confidentiality of their records when they interact with students [17, 27].
- The fear and reservations of individuals with and without willingness to participate should be addressed by providing information and obtaining informed consent [26, 41, 57].
- Written and verbal information about the training structures, the process, and the conditions should be provided [4, 26, 47].
- Practice guidelines can be planned and discussed with patients to address sensitive or stressful issues before training starts [29, 32].
- Patients should be informed about the skills and the level of student training [17].
- It is essential to create awareness of sensitive patient issues among practice supervisors and among students [35, 41, 56].
- It is recommended to talk “with” rather than “about” the patient and to either avoid or explain medical or nursing terminology [26, 31, 32].
- The duration of the learning unit should be planned and communicated to patients. This information helps clarify expectations and reduce frustration.
- Some patients, particularly severely ill patients and children, find participation boring. Therefore, the duration of a session should be adapted according to each individual [25, 29, 51].
- Smaller groups of up to four students were found to be more comfortable for most participants. This limit should be clarified when informed consent is obtained [22, 28, 29, 54].
- Patients should be actively involved in the teaching process and be given opportunities to discuss their experiences and evaluations [25, 41, 56].

Although the percentage of people who did not want to participate was low according to the studies identified here, this possibility should be considered by those responsible for training.

## Conclusions

This scoping review reveals that while patients generally accept and often value participation in clinical teaching, their experiences are substantially shaped by how the education is organized, communicated, and integrated into their care. Three conditions emerge as foundational to ethical, patient-centred clinical teaching: transparent communication about educational contexts and student roles; genuine informed consent processes that respect patient autonomy; and organizational approaches that protect privacy, limit the burden, and demonstrate respect for patient time and dignity.

However, a critical evidence gap emerged: all the identified research has examined medical education contexts, leaving nursing education without an empirical foundation for the development of evidence-based, patient-centred teaching practices. While some findings are likely transferred across health professions—particularly regarding informed consent principles and communication quality—the distinct nature of nursing care (sustained patient contact, intimate personal care activities, diverse care settings, and relationship-based practice) necessitates a nursing-specific investigation rather than uncritical extrapolation from medical education.

The current evidence gap represents not only an academic concern but also an ethical imperative. Nursing education’s commitment to patient-centred care requires that educational practices themselves embody this value by genuinely centring patient voices, preferences, and experiences.

The development of ethically sound, evidence-based approaches to patient involvement in nursing education requires dedicated research that examines patient experiences in nursing-specific contexts. Until such evidence is developed, nursing educators and institutions must proceed with particular ethical attentiveness, adapting available evidence thoughtfully while recognizing its limitations, implementing robust consent and feedback processes, and maintaining commitment to patient-centred practices even in educational contexts. The evidence gap identified in this review constitutes a clear research priority for nursing education scholarship, with direct implications for ethical practice and patient care quality.

## Supplementary Information

Supplementary Material 1.

## Data Availability

Not applicable. This is a review article and all data analyzed are derived from published sources, which are cited in the reference list.
