# Supplementary material for: Patient perspectives on practice guidance in nursing education: a scoping review
Source: BMC Med Educ. 2026 Mar 19;26:577. doi: 10.1186/s12909-026-09019-8 (PMC13064228; doi:10.1186/s12909-026-09019-8)
Supplement: Supplementary file 1 — Supplementary Material 1. [file 12909_2026_9019_MOESM1_ESM.doc]

**Complete search strategy**

***PubMed***

("patient*"[Title/Abstract] OR "inhabitant*"[Title/Abstract] OR "resident^*^"[Title/Abstract] OR "home occupant*"[Title/Abstract] OR "geriatric resident"[Title/Abstract] OR "sick person"[Title/Abstract] OR "Patients"[Mesh]) AND ("attitude"[Title/Abstract] OR "satisfaction"[Title/Abstract] OR "view"[Title/Abstract] OR "experience"[Title/Abstract] OR "benefit"[Title/Abstract] OR "acceptance"[Title/Abstract] OR "expectation*"[Title/Abstract] OR "involvement"[Title/Abstract] OR "participation"[Title/Abstract] OR "information"[Title/Abstract] OR "patient right*"[Title/Abstract] OR "consent"[Title/Abstract] OR "confidentiality"[Title/Abstract] OR "privacy"[Title/Abstract] OR "feel*"[Title/Abstract] OR "concern*"[Title/Abstract] OR "reservation*"[Title/Abstract] OR "motivation"[Title/Abstract] OR "incentive"[Title/Abstract] OR "informed consent"[Title/Abstract] OR "Patient Satisfaction"[Mesh] OR "Patient Participation"[Mesh] OR "Motivation"[Mesh] OR "Patient Rights"[Mesh]) AND ("Bedside teaching"[Title/Abstract] OR "teaching round*"[Title/Abstract] OR “clinical teaching*"[Title/Abstract] OR "hands on learning"[Title/Abstract] OR "Teaching Rounds"[Mesh])

**Filter**: Languages: German and English (28 results)

**Results as of August 9, 2022:** 1255

***CINAHL***

(TI("patient*" OR "inhabitant*" OR "resident^*^" OR "home occupant*" OR "geriatric resident" OR "sick person") OR AB("patient*" OR "inhabitant*" OR "resident^*^" OR "home occupant*" OR "geriatric resident" OR "sick person") OR MH("Patients" OR "Nursing Home Patients")) AND (TI("attitude" OR "satisfaction” OR "view” OR "experience" OR "benefit" OR "acceptance" OR "expectation*" OR "involvement" OR "participation" OR "information” OR "patient right*" OR "consent" OR "confidentiality" OR "privacy" OR "feel*" OR "concern*" OR "reservation*" OR "motivation" OR "incentive" OR "informed consent") OR AB("attitude" OR "satisfaction” OR "view” OR "experience" OR "benefit" OR "acceptance" OR "expectation*" OR "involvement" OR "participation" OR "information” OR "patient right*" OR "consent" OR "confidentiality" OR "privacy" OR "feel*" OR "concern*" OR "reservation*" OR "motivation" OR "incentive" OR "informed consent") OR MH("Patient Satisfaction" OR "Patient Rights" OR "Privacy and Confidentiality" OR "Motivation")) AND (TI("Bedside teaching" OR "teaching round*" OR “clinical teaching*" OR "hands on learning") OR AB("Bedside teaching" OR "teaching round*" OR “clinical teaching*" OR "hands on learning") OR MH("Teaching Methods, Clinical"))

**Filter**: Languages: German and English (31 results)

**Results as of August 9, 2022:** 647. Deleted duplicates from CINAHL: 5. (see: https://connect.ebsco.com/s/article/How-does-EBSCO-prevent-duplicate-results-from-appearing-in-the-search-results-list?language=en_US)

***Livivo – ZB MED Köln***

FS=(patient* OR inhabitant* OR resident^*^ OR "home occupants" OR "geriatric resident" OR "sick person" OR patient OR inhabitant OR "home occupant" OR Kranke OR Kranker OR Pflegebedürftige OR Pflegebedürftiger OR Bewohner OR Bewohnerin OR "zu pflegende Person" OR "kranke Person") AND (FS=(attitude OR satisfaction OR view OR experience OR benefit OR acceptance OR expectation* OR expectation OR involvement OR participation OR information OR "patient right" OR "patient rights" OR consent OR confidentiality OR privacy OR feel OR feel* OR concern* OR concern OR reservation* OR reservation OR motivation OR incentive OR "informed consent" OR Einstellung OR Zufriedenheit OR Wohlbefinden OR Ansicht OR Sichtweise OR Sichtweisen OR Erfahrung OR Erfahrungen OR Nutzen OR Vorteil OR Akzeptanz OR Erwartungen OR Beteiligung OR Teilnahme OR Information OR Informationen OR Patientenrecht OR Patientenrechte OR Zustimmung OR Erlaubnis OR Vertraulichkeit OR Privatsphäre OR Datenschutz OR Gefühle OR Gefühl* OR Bedenken OR Vorbehalte OR Reaktion OR Reaktion* OR Integration OR integrieren OR Motivation OR Motivation* OR Anreiz OR "informierte Zustimmung" OR "Einwilligung nach Aufklärung")) AND (FS("Bedside teaching" OR "teaching round" OR "clinical teaching" OR "hands on learning" OR "Unterricht am Krankenbett" OR Anleitung OR Praxisanleitung OR "pflegerische Anleitung" OR "Unterricht am Patientenbett"))

**Results as of August 9, 2022:** 11
